# Acknowledgement to Reviewers of *Marine Drugs* in 2019

**DOI:** 10.3390/md18010068

**Published:** 2020-01-20

**Authors:** Marine Drugs Editorial Office

**Affiliations:** MDPI, St. Alban-Anlage 66, 4052 Basel, Switzerland

The editorial team greatly appreciates the reviewers who have dedicated their considerable time and expertise to the journal’s rigorous editorial process over the past 12 months, regardless of whether the papers are finally published or not. In 2019, a total of 723 papers were published in the journal, with a median time to first decision of 13 days and a median time from submission to publication of 32 days. The editors would like to express their sincere gratitude to the following reviewers for their generous contribution in 2019:
Aarstad, Olav AndreasLorenzo, Jose M.Abdelmohsen, Usama RamadanLorenzo-Morales, JacobAbdollahi, MehdiLoret, Erwann P.Aberturas, JavierLouis, SandrineAbeywardana, TharindumalaLouzao Ojeda, M. CarmenAbugri, Daniel A.Lowell, AndrewAcquaviva, RosariaLu, JunAdamovsky, OndrejLuckenbach, TillAdams, Bryn L.Ludwiczuk, AgnieszkaAdamus-Białek, WiolettaLuvisetto, SiroAdema, CoenLyakhova, Ekaterina G.Adunyah, Samuel E.Lyukmanova, Ekaterina N.Afiyatullov, Shamil Sh.Ma, WanshuAflalo, ClaudeMacedo, AngelaAfonso, Clélia N.Maciejczyk, MateuszAgarwal, VinayakMaciel, ElisabeteAgrawal, Samir G.Madhukar, BurraAguilera Correa, John JairoMadhyastha, RadhaAiello, GildaMaeda, HayatoAkagi, YukiMaeda, YuichiAkins, RobertMaehre, HanneAlbergamo, AmbroginaMaffei, AngeloAldred, NicholasMaggi, Leonard B.Al-Horani, Rami A.Makarieva, TatyanaAli, RameezMalik, RavinderAl-Mourabit, AliMallis, PanagiotisAlves, ElianaMaltseva, ArinaAmillis, SotirisMalyarenko, Olesya S.Aminin, Dmitry L.Mammadova-Bach, ElminaAnand, PrachiManakhov, AntonAncheeva, ElenaMancini, InesAndersson, Håkan SMándi, AttilaAndo, YoshitoMania, SzymonAndolfi, AnnaMankowski, MarkAndrade, Paula B.Mann, FrancisAndreev, KonstantinManville, RíanAndreotti, GiuseppinaMarcus, Schmitt-EgenolfAndrianasolo, EricMargassery, Lekha MenonAnedda, RobertoMari, MicheleAnraku, MakotoMarin, LuminitaAntiga, EmilianoMarinov, Georgi K.Antolak, HubertMariottini, Gian LuigiArana, InesMárki, ÁrpádAraniciu, CătălinMarroqui, LauraAraóz, RómuloMartí, SergioArita, MinetaroMartín-Aragón, SagrarioArmanini, DecioMartinez, RonnyArnich, NathalieMartínez-Alvarez, OscarArtini, MarcoMartínez-del-Pozo, AlvaroAsai, DaisukeMartínez-Espinosa, Rosa MaríaAtashrazm, FarzanehMartín-Gil, JesusAugustyniak, DariaMartini, ClaudiaAvila-Román, JavierMartinotti, SimonaAwakawa, TakayoshiMartins, EvaAyciriex, SophieMartins, RosarioAzam, LaylaMartins, SoniaBacchiocchi, SimoneMarverti, GaetanoBae, Hyung D.Marzocco, StefaniaBaglieri, JacopoMasłyk, MaciejBai, RenrenMasullo, MariorosarioBaindara, PiyushMateo, CesarBaker, BillMati-Baouche, NarimaneBaker, YsobelMatkowski, AdamBakowsky, UdoMatsugo, SeiichiBalabanova, LarissaMatsumiya, MasahiroBanoub, Joe HMatsuura, HiroshiBarbaglio, AliceMavila, SudheendranBardakova, Kseniia N.Mavridis, KonstantinosBardi, GiuseppeMavrogonatou, EleniBarja, Juan L.Máximo, PatríciaBarnathan, GillesMay, AaronBarreca, DavideMazur-Bialy, Agnieszka IrenaBarry, Amanda N.Mazur-Marzec, HannaBasini, GiuseppinaMcCormick, AlistairBatista, IrineuMcDermid, Karla J.Baumann, MarcusMcKinnie, Shaun M.K.Bayascas, Jose RMcPhail, Kerry L.Bazire, AlexisMcRose, DarcyBednarek, RadoslawMehbub, Mohammad FerdousBedoux, GillesMehiri, MohamedBeemelmanns, ChristineMeier, Raphael P.H.Behringer, ErikMelo, TâniaBeld, JorisMemo, MaurizioBello, ClaudiaMenaa, FaridBellstedt, PeterMéndez, LucíaBeltowski, JerzyMenzel, LorenzoBenedetti, ManuelMerah, OthmaneBéni, SzabolcsMeschwitz, SusanBennett, Alan B.Meyerholz, David K.Berdyshev, Evgeny V.Mi, Fwu-LongBerezovski, Maxim V.Miki, YasuhiroBerger, StefanMilewski, SławomirBergès, ThierryMilisenda, GiacomoBerillis, PanagiotisMilledge, John J.Berlinck, RobertoMiller Conrad, LauraBernardes, NunoMills, KerryBerrue, FabriceMimaki, YoshihiroBertin, Matthew J.Minami, AkiraBessa, LucindaMinkiewicz, PiotrBhattacharya, SomanonMishra, NigamBianco, ArmandodorianoMitaine-Offer, Anne-ClaireBiedermann, DavidMitchell, CarterBiernasiuk, AnnaMitchell, MiguelBijak, MichałMiyaoka, HiroakiBikiaris, DimitriosMiyashita, KazuoBingham, Jon-PaulMočibob, MarkoBirbeck, Johnna A.Mogielnicki, AndrzejBišová, KateřinaMoh, Sang HyunBitra, ArunaMolgó, JordiBlanco, JuanMolinaro, AntonioBlanco, MariaMolinski, TadeuszBlasiak, JanuszMöller, LucianaBlombach, BastianMollica, AdrianoBlumer-Schuette, Sara E.Mollo, ErnestoBoddy, Christopher NMonastyrnaya, MargaritaBodin, JohannaMoncalián, GabrielBoekema, BoukeMonchaud, DavidBogni, AlessiaMoni, LisaBøgwald, JarlMontenegro, IvánBoix, EsterMonti, Maria ChiaraBonadies, IreneMora, LeticiaBonamore, AlessandraMorabito, RossanaBoncela, JoannaMoreno Gonzalez, InesBond, Simon TMorganti, PierfrancescoBondon, ArnaudMori, TetsushiBoothello, RioMorikawa, ToshioBorbás, AnikóMorimura, ShigeruBorradaile, NicaMorita, NaokiBorsetto, ChiaraMorris, GordonBoryski, JerzyMorse, DavidBossi, FleurMortara, LorenzoBoto, AliciaMorzycki, Jacek W.Böttcher, ThomasMosqueira, MatiasBouaïcha, NoureddineMoura, Maria J.Bourdelais, AndreaMouradov, AidynBourguet-Kondracki, Marie LiseMourão, Paulo A.S.Boutcher, YatiMourelle, M. LourdesBovio, ElenaMozuraityte, RevilijaBowden, BruceMukai, HidehitoBoysen, ReinhardMüller, ChristophBracher, FranzMüller, JoachimBradshaw, BenMüller, Werner E.G.Bradshaw, NicholasMulloy, BarbaraBrandenburg, KlausMurai, NoriyukiBrault, Jeffrey J.Murakami, KazumaBrecker, LotharMuramoto, KojiBresson-Bepoldin, LaurenceMurkin, AndrewBrinchmann, Monica FengsrudMurphy, Patrick J.Broadhurst, C. LeighMuschiol, JanBross, PeterMusioł, RobertBrown, Christopher LyonMusso, LoanaBrown, LindsayMusso, OrlandoBrownlee, Iain AMuthuramu, IlayarajaBrück, ThomasMyszka, KamilaBrunel, Jean MichelNagai, HiroshiBrycki, BogumilNagamine, TakeakiBubnova, EkaterinaNagarajan, BalajiBucolo, ClaudioNajdenski, HristoBudny, MarcinNakagomi, TakayukiBugni, TimNakamura, MotokiBukowski, MichalNakatani, YoshihikoBullon, PedroNam, Kyung SooBurcul, FrankoNam, Sang-JipBurke Da Silva, KarenNanjappa, DeepakByun, KyungheeNash, KevinCabral, JaydeeNawrot, BarbaraCacciotti, IlariaNazarski, Ryszard B.Cacho Teixeira, MiguelNedoluzhko, ArtemCadamuro, MassimilianoNencioni, LuciaCaetano, NídiaNett, MarkusCaffrey, PatrickNeumann, UlrikeCakstina, IneseNevzorova, Yulia A.Calado, Maria Da LuzNewman, David J.Calado, RicardoNguyen, Ba Thuy LinhCalcabrini, CinziaNguyen, Thanh BinhCalcutt, MichaelNi, HongminCaldara, MarinaNicolai, MarisaCalero Valdayo, Patricia MariaNicolantonio, D’OrazioCallery, Patrick S.Nicoletti, RosarioCalvo, LourdesNielsen, Vance GirardCampbell, ChristopherNifantiev, NikolayCampiglia, PietroNishimura, ShinichiCankar, KatarinaNishiwaki, HisashiCao, Jay J.Nofer, Jerzy-RochCapon, RobNonogaki, KatsunoriCaputo, GregoryNorte, ManuelCarballal, Ramon NovoaNoshita, ToshiroCardullo, NunzioNovick, DanielaCarlisi, DanielaNurtdinov, RamilCaro, YanisNuzzo, GenoveffaCarocho, Márcio SoaresNycz, JacekCarradori, SimoneO’Loughlin, Colleen T.Caruso, GabriellaObanda, DianaCarvalho, PauloO’Doherty, GeorgeCasapullo, AgostinoOfir, RivkaCastro, José Javier FernándezOgórek, RafałCastro, María ÁngelesOh, Dong-ChanCatanzaro, ElenaOh, JaehyeonCatarino, Marcelo D.Oh, Ki-BongCatto-Smith, AnthonyOh, Won KeunCeranowicz, PiotrO’Harte, FinbarrCerra, BrunoOhashi, NamiCertal, Ana CatarinaOhman, Dennis E.Chae, HeeyoungOhno, OsamuChang, Yu-WeiOhshiro, TakashiChankhamjon, PranatchareeyaOhta, ShinjiCharoensiddhi, SuvimolO’Keefe, BarryChauhan, NeerajOkunade, Adewole L.Chaves, SílviaOlejnik, AnnaChedea, VeronicaOliveira, AndreiaChen, Jiann-ChuOliveira, CatarinaChen, LihuaOliveira, Paula A.Chen, Rong-JaneOliviero, GiorgiaCheng, Juei-TangOmidian, KosarCheng, Meng-ChunOrtega, María JesúsChetwynd, AndrewOsborn, HelenChevrot, RomainOsorio, CarlosChhabra, GaganOspina-Millán, Claudia A.Chianese, GiuseppinaOstrowska, KingaChiesa, EnricaOthman, Eman M.Chiesa, GiuliaOuazzani, JamalCho, JungsookOvchinnikova, Tatiana V.Cho, Young-EunOwen, JeremyChobot, VladimírOzaki, TaroChoi, HyukjaeOzaltin, KadirChoi, Yeung JoonOzeki, YasuhiroChoi, Yung-HyunPace, VittorioChollet-Krugler, MarylènePaetz, ChristianChoshi, TominariPagano, StefanoChoudhary, HemantPalanisamy, Satheesh KumarChrapusta-Srebrny, EwelinaPalenzuela López, José AntonioChristopher W., KirbyPallottini, ValentinaChronopoulou, EvangeliaPalma, Paulo JChuang, Ta-HsienPalmer, AndrewChung, Byung YeoupPalmer, MichaelChyau, Charng-CherngPandya, Anshul A.Cicciù, MarcoPang, BoCicero, ArrigoPang, Ka-LaiCirrincione, GirolamoPanzarini, ElisaCiufolini, Marco A.Panzella, LuciaCivera, Maria ConcepciónPapapolymeroua, Georgios A.P.Clanchy, FelixParé, Paul W.Clark, MelodyParikesit, Arli AdityaClericuzio, MarcoParisini, EmilioCocca, EnnioPark, Hee-SooCoccheri, SergioPark, Hyeung-geunCochis, AndreaPark, JoshuaColeman, Mitchell C.Park, SangkyuColla, Luciane MariaPark, Shin-HyungCollier, JackieParolini, CinziaColnaghi, LucaParrilli, ErmenegildaConcas, AlessandroParrino, BarbaraCongestri, RobertaPatarrão, RitaConti, PioPatel, NibeditaCooke, Ira R.Pathak, HarshCoombs, MelaniePatruno, AntoniaCopolovici, Dana MariaPatruno, MarcoCorsaro, Maria MichelaPau-Roblot, CorinneCosta de Morais, Rui ManuelPavon-Djavid, GracielaCosta, FabíolaPeana, Massimiliano F.Costantino, LucaPearce, CedricCostantino, ValeriaPeigneur, SteveCosta-Pinto, Ana RitaPeleteiro, MercedesCox, Jonathan A.G.Pelin, MarcoCroaker, AndrewPeltomaa, ElinaCrossman, LisaPennock, NathanCrüsemann, MaxPepi, MilvaCsuk, RenéPereira, José AugustoCuellar, SaraPereira, LeonelCueto, MercedesPerez Pascual, DavidCui, ZhengPerin, GiorgioCumming, Mathew HoaniPeriyasamy, PalsamyCutignano, AdelePerrier, JosetteCzjzek, MirjamPersico, MarcoCzyż, KatarzynaPerteghella, SaraD’Anneo, AntonellaPetek, SylvainD’Errico, StefanoPeter, MartinDabrowski, JanuszPeterson, Eric CharlesD’Agostino, PaulPetit, Patrice X.Dahiya, RajivPetrosino, MariaDalla Pozza, ElisaPhan, Chin-SoonDaly, NorellePhelan, VanessaDamiani, GiovanniPhelix, Clyde F.D’Amora, UgoPiccialli, VincenzoDandawate, PrasadPickering, RaeleneDannaoui, EricPickova, JanaDansette, Patrick M.Picot, LaurentDaranas, Antonio HernándezPiegat, AgnieszkaDarbin, Olivier EricPiemontese, LucaDas, UndurtiPierri, Ciro LeonardoD’auria, ValeriaPiggott, AndrewDavinelli, SergioPina Pérez, Maria ConsueloDawson-Scully, KenPinto, Diana CláudiaDaza Navarro, PaulaPiprek, Rafal P.De Agostini, ArianePiras, Anna MariaDe Blasio, AnnaPires, CarlaDe Jesus Raposo, Maria FilomenaPisapia, PasqualeDe La Calle, FernandoPitingolo, GabrieleDe La Luz Garcia-Hernandez, MariaPitsinos, EmmanuelDe Marco, ArioPlanas, AntoniDe Martin, SaraPlastina, PierluigiDe Pascale, DonatellaPlatta, HaraldDe Paz, Jose LuisPlavec, JanezDe Souza, CatharinaPogorzelska, AnetaDean, AndrewPohl, SebastianDeb, SubrataPoirier, DonaldDebitus, CecilePoku, Rosemary A.Dedos, Skarlatos G.Polen, TinoDefoirdt, TomPoma, PaolaDegl’Innocenti, DonatellaPoniedziałek, BarbaraDel Giudice, RitaPopolo, AdaDelbarre Ladrat, ChristinePotopnyk, MykhayloDéleris, PaulPotterat, OlivierDella Greca, MarinaPoudel, Tej NarayanDella Sala, GerardoPoulin, Remingtonden Hertog, JeroenPozo, ÓscarDeng, HaiPozzolini, MarinaDenny, BillPrado-Cabrero, AlfonsoDerreumaux, PhilippePrakash, DivyaDhakal, DipeshPratsinis, HarrisDi Bari, LorenzoPrentice, HowardDi Donato, PaolaPrentis, PeterDi Maro, AntimoPrieto, LauraDiana, PatriziaProkai, LaszloDias de Almeida, M. CatarinaProkopov, TsvetkoDias, RicardoProksch, PeterDiaz De Cerio, OihanePsurski, MateuszDiaz-Marrero, Ana R.Pucciarelli, SandraDijkstra, BaukePuglisi-Weening, MelanyDilokpimol, AdipholPuillandre, NicolasDinos, GeorgiosPuppa, MelissaDjerada, ZoubirPytkowska, KatarzynaDoda, Sai ReddyQuaglino, DanielaDoddapattar, PrakashRabbani, GulamDoligalska, MariaRadaram, BhaskerDomingues, RosarioRadošević-Stašić, BiserkaDomingues, SaraRagg, EnzioDomínguez Álvarez, EnriqueRahimi, FaridDomínguez, HerminiaRahman, AzizurDominguez, Juan CarlosRai, DilipDonato, Rosario FrancescoRajauria, GauravDong, ShihuiRakotondraibe, Harinantenaina LivaDormond, OlivierRämä, TeppoDorotkiewicz-Jach, AgataRamakumar, KinthadaDosoky, NouraRamaraj, PanduranganDouglas, TimothyRamirez-Vick, JaimeDoyen, AlainRamos, CarmenDrago, SilvinaRamos-Jiménez, ArnulfoD’Souza, RoshanRanzato, EliaDu, YongleRasid, OrhanDucret, MaximeRasinger, Josef D.Dudas, JozsefRateb, Mostafa E.Duddupudi, AnanthaRather, GulamDufossé, LaurentRauch, BernhardDufour, AntoineRaugei, GiovanniDuggan, Brendan M.Razani, BabakDuh, Chang-YihRebuffat, SylvieDumic, JerkaReddy, PriyankaDurán-Riveroll, LorenaReeve, VivienneĐurović, SašaRehel, KarineD’Urso, AlessandroReimann, Regina R.Dutertre, SebastienReitzel, AdamDutot, MélodyRemesh, SoumyaDvořáčková, MartinaRemigante, AlessiaDyshlovoy, Sergey A.Renaud, JustinDzhemileva, LilyaRennert, ChristianeEbada, SherifResende, Diana I.S.P.Ebrahim, WeaamRevuelta, JuliaEckl, PeterReyes, FernandoEgami, HiromichiRhee, Jin-KyuEhrlich, HermannRho, Jung-RaeEisenberg, TobiasRibchester, RichardEkker, MarcRiccio, GennaroEl Khalloufi, FatimaRiccio, RaffaeleEl Midaoui, AdilRigano, DanielaEldeeb, MohamedRinge, DagmarEl’kin, Yu. N.Rishi, GautamEllinger, BernhardRivas, FatimaEl-Moghazy, AhmedRizzolio, FlavioEl-Sayed, IbrahimRobertson, Mark J.El-Seedi, HeshamRobles, LukeElshahawi, SherifRoca, MaríaElst, KathyRodrigues Reis, Cristiano EduardoEmmanouela, FilippidiRodríguez, Armando AlexeiEppink, MichelRodríguez, JaimeEscargueil, AlexandreRodríguez-Pérez, CeliaEsteve-Romero, JosepRogato, AlessandraEvanno, LaurentRohde, John R.Evans, Franklin D.Romano, GiovannaEvin, GenevièveRomano, StefanoFabris, MicheleRonsard, LaranceFakira, Amanda KRoss, Ian LFalconer, Ian R.Rossi, AdrianoFanizzi, Francesco PaoloRossi, SilviaFauzi, IlianaRossignol, JulienFedoreyev, SergeyRouge, PierreFenical, WilliamRoussis, VassiliosFernandes, Carla Sofia GarciaRowley, DavidFernández, José J.Roy, AnuradhaFernandez-Martinez, LorenaRóżalska, BarbaraFernández-Ochoa, ÁlvaroRubino, Federico MariaFerrari, PaolaRubio, MyriamFerreira, Paula M.Ruiz Téllez, TrinidadFerrie, SuzieRupasinghe, ThusithaFerrieres, VincentRusnati, MarcoFesta, CarmenRusso, PatriziaFiedler, Hans-PeterRussu, WadeFigiel, AdamRustad, TuridFiguerola, BlancaRutherford, StevenFilker, SabineRuvio, RaquelFitton, HelenRyan, AliFlorczyk, StephenRybak, Leonard P.Floresta, GiuseppeRyu, BoMiFlórez-Fernández, NoeliaRzymski, PiotrFocaroli, StefanoSabatier, Jean-MarcFogacci, FedericaSabila, PaulFontana, AngeloSadowska, BeataFonteh, Alfred N.Saez, CarmenFontés, MichelSafavi, HelenaFouillaud, MireilleSagnou, MarinaFousteris, ManolisSaha, Sushanta KumarFrancavilla, MatteoSahariah, PriyankaFranco, ChrisSaid, NeveenFranken, Nicolaas A.P.Saito, SatoshiFratini, FilippoSakai, TakeoFretté, XavierSakamoto, ToshioFrisch, BenoîtSakilam, SatishFrkanec, RužaSala, RobertoFroldi, GuglielminaSalas, JoseFrosini, MariaSalim, AngelaFruttero, Leonardo L.Samhan-Arias, AlejandroFu, WeiqiSan Juan Serrano, FuencislaFujita, MasakiSanchez-Amat, AntonioFujita, MorifumiSánchez-García, DavidFujita, YukiSanchez-Martinez, MelchorFumagalli, LauraSanjeewa, Kalu Kapuge AsankaFunabara, DaisukeSanjust, EnricoFuqua, Joshua LSansone, ClementinaGaascht, FrancoisSantabarbara, StefanoGacesa, RankoSantos, Juan C.Gaffet, EricSantos-Ruiz, LeonorGajjar, ChiragSantucci, AnnalisaGalanis, AlexisSapudom, JiranuwatGalasiti Kankanamalage, AnushkaSarabia, FranciscoGalasso, ChristianSarapultsev, AlexeyGaldiero, MassimilianoSarkar, DhrubaGales, LuísSarmad, ShokatGanesan, A.Satake, MasayukiGarbayo, InésSato, EiichiGarcia Vaquero, MarcoSato, NorihiroGarcia, Coralia V.Sato, ShinGarcia, FaynaSatomi, YoshikoGarcia-Pardo, JavierSaurav, KumarGarg, NehaSavatin, Daniel-ValentinGarikipati, DilipSaviola, AnthonyGarinis, GeorgeSavion, NaphtaliGarrido, AntonioSaw, Constance L.L.Gaspar, DianaScalise, MariafrancescaGaspar, HelenaScapagnini, GiovanniGasparri, FabioScarfì, SoniaGasparro, RobertaSchmalz, Hans-GuentherGasparski, AlexanderSchmidt, MartinGatesy, John E.Schmidtke, GunterGatti, LauraScholls, DominiqueGebauer, JanSchröder, Heinz C.Gęgotek, AgnieszkaScoffone, Viola CamillaGemmill, TrentSearcey, MarkGenovese, GiuseppaSeca, Ana Maria Loureiro DaGerdol, MarcoSekiya, MizukiGerothanassis, Ioannis P.Semino, CarlosGesell, AndreasSeo, Ho SeongGiampietro, LetiziaSerra, DolorsGiannini, CleliaSerra, StefanoGilabert-Oriol, RogerServent, DenisGiordano, AntonioShabunin, Anton S.Giovannoni, RobertoShakya, Viplendra Pratap SinghGirelli, Chiara RobertaShang, ZhuoGismondi, AngeloShankar, EswarGiuffrida, DanieleSharma, Bhesh RajGiunchedi, PaoloShavandi, AminGkelis, SpyrosShekhova, ElenaGlaser, KeithSheldrake, GaryGliszczynska, AnnaShikov, AlexanderGoel, Peeyush N.Shim, SehwanGolokhvast, KirillShimasaki, ToshiakiGomes, NelsonShin, Hee JaeGómez, Ana MaríaShin, JongheonGoncalves, OlivierShirahata, TatsuyaGonec, TomasShishido, Tânia K.Gong, XiaomingShnyder, SteveGonkowski, SlawomirShornick, LaurieGorbi, StefaniaShoshan, MariaGoto-Inoue, NaokoShurpik, DmitriyGotsbacher, MichaelSiciliano, CarloGoua, MarieSiebert, Hans-ChristianGoudoulas, ThomasSikazwe, DonaldGozzo, FrancoSil, PayelGradinaru, Andrei CristianSilchenko, Artem S.Grauso, LauraSilipo, AlbaGraziano, GuellaSilkina, AllaGreco, ClaudioSilva, MarceloGreen, Paul W CSilva, Pedro JorgeGrisk, OlafSilva, Simone S.Grkovic, TanjaSilvestre, Armando J. D.Grochowski, CezarySimões, Marta FilipaGrogan, GideonSimpson, Thomas J.Gross, Joshua B.Singh, AnandGrottesi, AlessandroSingh, JonathanGuerra, José Antonio GuerraSingh, KamalendraGuerrero, Miguel G.Singh, RajbirGupta, KajalSingh, ReemaGurevich, Vsevolod V.Singh, SonalGuriec, NathalieSingla, BhupeshGustafson, KirkSiodłak, DawidGutiérrez-Gallego, RicardoSkalko-Basnet, NatasaHaass, Nikolas K.Skellam, ElizabethHabala, LadislavSkidmore, MarkHaertle, ThomasSkorik, Yury A.Hagi, TatsuroSkouta, RachidHaines, Julie ReiszSmith, DavidHallegraeff, GustaafSmith, Geoffery JasonHamaguchi, MasahideSmith, Thomas E.Han, Dong-WookSmith, Zacharias J.Han, Jae HongSmułek, WojciechHan, Jong WonSnell, Terry W.Han, Se JongSogawa, ChiharuHanif, NovriyandiSolano, FranciscoHansen, EspenSolovchenko, AlexeiHanski, LeenaSon, Kwang-heeHarder, JensSosa, SilvioHardiman, GarySoto, JulioHarijan, Rajesh KSotomayor, Camilo G.Harms, HenrikSoulère, LaurentHarvey, Patricia J.Souto, María LuisaHarvey, PetaSpeciale, AntonioHasan, Mohammad H.Sprawka, IwonaHasche, DanielSpurio, RobertoHashimoto, MakotoSreerama, SubramanyaHashimoto, MasaruSrivastava, SaurabhHaug, TorSrivedavyasasri, RadhakrishnanHelaly, SoleimanSroka, ZbigniewHelesbeux, Jean-JacquesStabili, LoredanaHeneberg, PetrStacewicz-Sapuntzakis, MariaHenkel, ChristiaanStachowicz, AnetaHenry, Ryan A.Starek, MałgorzataHeo, Soo-JinStender, Emil G. P.Hepnarova, VendulaStevens, David ColeHermund, Ditte B.Stoikov, Ivan IvanovichHildreth, BlakeStoklosa, RyanHiltunen, MinnaStoner, ElizabethHindra, HindraStonik, ValentinHinds, Terry D.Stoskopf, MichaelHirakawa, HidehikoStuhr, MarleenHirasaka, KatsuyaSubramaniam, PrasadHoang, Nam VSuckling, Colin J.Holguin, Francisco OmarSujak, AgnieszkaHomma, TakujiroSuleria, Hafiz Ansar RasulHonecker, FriedemannSumi, ShoichiroHong, KuiSurguchov, AndreiHong, Soon-KwangSvendsen, Morten Bo SøndergaardHore, Michael J. A.Svetashev, VasilyHossain, Md EkhtearSykłowska-Baranek, KatarzynaHovde, Blake T.Synytsya, AndriyHsieh, YvesSytykiewicz, HubertHsu, Fang RongSzablewski, LeszekHsu, Hsien-YehSzakonyi, ZsoltHuang, Chun-YungSzepanowski, FabianHuang, Tse-HungSzewczyk, KatarzynaHuber, Eva M.Sztanke, MałgorzataHudson, David M.Szweda, PiotrHung, AndrewSzymańska, EmiliaHungerford, JamesTabudravu, Jioji N.Hwang, HayoungTada, RuiHwang, Long-ChihTae, Han-ShenIanora, AdriannaTagliazucchi, DavideIatrou, HermisTailhades, JulienIbeanu, GordonTaira, JunseiIbrahim, FandiTajiri, NaokiIijima, NoriakiTakahashi, NobuyukiIlaš, JanezTakaichi, ShinichiImhoff, JohannesTakao, OjimaImperatore, ConcettaTakemori, HiroshiInuki, ShinsukeTakemura, MihoInuzuka, ToshiyasuTalora, ClaudioIoannou, EfstathiaTaly, AntoineIop, LauraTamura, Jun-ichiIorga, Bogdan I.Tanabe, KenjiIqbal, Hafiz M. N.Tanaka, JunichiItabashi, YutakaTanaka, NaonobuIto, YoichiroTanaka, SatoshiItoh, TakafumiTaniguchi, TohruIvancic Santek, MirelaTarantino, GiovanniIwasaki, ArihiroTasdemir, DenizJackson, Stephen A.Tashima, ToshihikoJain, PrashantTava, AldoJaiswal, Ashvin R.Tavío, María M.Jallet, DenisTawfike, AhmedJanczarek, MonikaTayebati, Khosrow SayedJanda, ElzbietaTaylor, SteveJang, SehwanTebben, JanJanganan, ThamaraiTecilla, PaoloJanouškovec, JanTenorio, Manuel JimenezJanowski, MiroslawTeodoro, JoãoJansen, RolfTerada, TetsuyaJaradat, NidalTerme, NolwennJaskiewicz, MaciejTeta, RobertaJaźwiński, JarosławTheoduloz, CristinaJelonek, KatarzynaThiel, TeresaJenner, RonaldThil Smith, Adam AlexanderJennings, Jessica AmberThinon, EmmanuelleJeon, You-JinThirunavukarasu, DeepakJeong, Se KyooThomas, Carole D.Jia, YingThomas, OlivierJimbo, MitsuruTidgewell, KevinJimenez, CarlosTietjen, IanJiménez-Barbero, JesusTietze, AlesiaJoly, FrancineTietze, DanielJónsson, Zophonías O.Tikhonov, VladimirJordao, LuisaTimashev, PeterJuan Fernando, Martinez-LealTimoshenko, AlexanderJung, JeeTiriveedhi, VenkataswarupJung, wonkyoTischler, DirkKaas, QuentinTiwari, PoojaKaczmarek, BeataTiwari, Rakesh K.Kadokawa, Jun-ichiTkaczewska, JoannaKafarski, PawełToepel, JörgKageyama, HakutoTomaszewska, EwaKalinin, VladimirTomoda, HiroshiKamiya, MitsunobuTorikai, KoheiKamlage, BeateTornesello, Anna LuciaKanakkanthara, ArunTorres, Carlos F.Kaneko, GenTorres, María DoloresKang, HeonjoongTørris, ChristineKang, In-SungTossi, AlessandroKang, Jeong-HunTosti, ElisabettaKang, JongTownsend, EleanorKang, WonkuToyoda, MasashiKarayiannakis, AnastasiosTrainer, VeraKarentz, DenebTran, TrongKarmakar, ParthaTribulova, NarcisKarpinski, Tomasz M.Trincone, AntonioKaruso, PeterTrotsko, NazarKasai, FumioTsai, Ang-ChenKashman, YoelTsai, Jen-NingKasten-Jolly, JaneTsatsakis, AristidisKatada, KazuhiroTseng, Boo ShanKatikou, PanagiotaTsetlin, Victor I.Kato, HirohitoTsopmo, ApollinaireKato, Takamitsu A.Tsoupras, AlexandrosKavita, KumariTsurkan, Mikhail V.Kawai, ShigeyukiTuccinardi, TizianoKawamura, AkiraTudela, JoseKawashima, HidekiTurk, TomKayumov, AiratTurner, AndrewKellenberger, StephanTurrini, EleonoraKelly, MicheleTyśkiewicz, RenataKem, William R.Tziveleka, Leto AikateriniKerch, GarryTzovenis, IoannisKerr, RussellUdumula, Venkata ReddyKesharwani, SiddharthUeno, MikinoriKeyzers, RobUlber, RolandKhadka, ManojUmerska, AnitaKhalifa, ShadenUmeyama, AkemiKhalil, ZeinabUnno, TatsuyaKhan, MohsinUnudurthi, SathyadevKhozin-Goldberg, InnaUrban, PhilippeKhurshid, ChrowUrban, SylviaKieliszek, MarekUrbatzka, RalphKikuchi, TakashiUsami, YoshihideKim, HangunUstyuzhanina, NadezhdaKim, Hyeung-RakUwai, KojiKim, LouV Ivanov, AlexanderKirillova, AlinaVafidis, DimitrisKirsch, Gilbert H.Vairetti, MariapiaKiselevskiy, MikhailVale, CarmenKiuru, PaulaVale, PauloKleinegris, DorindeValentao, PatriciaKlejdus, BorivojVallesi, AdrianaKlettner, AlexaVamanu, EmanuelKlimov, Dmitry I.Van Der Merwe, MarieKnechtle, BeatVan Dyke, Michael W.Knudsen, GabrielVan Hees, StijnKo, Sung-kyunVan Wezel, GillesKobeissy, FirasVandooren, JenniferKocher, Thomas D.Vann, WillieKoga, Jun-ichiroVanthuyne, NicolasKogovšek, TjašaVarese, Giovanna CristinaKoike, KenzoVasas, GáborKokel, DavidVasconi, MauroKokkola, TarjaVavitsas, KonstantinosKoksharova, Olga A.Vázquez, José AntonioKolobov, Alexandr AlexandrovichVecino, XanelKolosnjaj-Tabi, JelenaVeenman, LeoKomaki, HisayukiVeerasikku Gopal, DeepaganKomba, ShiroVeeravalli, VijayabhaskarKong Thoo Lin, PaulVellecco, ValentinaKonno, KatsuhiroVelu, Sadanandan E.Kontek, BogdanVenus, JoachimKorbee-Peinado, NathalieVerdes, AidaKoschella, AndreasVerdon, JulienKoshino, HiroyukiVergara, DanieleKostas, Emily T.Verma, SurbhiKostecka, MalgorzataVerpeut, JessicaKostomitsopoulos, Nikolaos G.Vetvicka, VaclavKotoku, NaoyukiVieira, HelenaKoulman, AlbertVigani, BarbaraKovacs, RenatoVignes, MichelKoval, AlexeyVil’, Vera A.Kowalczewski, PrzemysławVilgis, ThomasKoyama, NobuhiroVinogradov, EvgueniiKozlov, SergeyVishe, MaheshKranzler, ChanaVladimir, KalininKrisch, JuditVlashi, ErinaKrishnakumar, DevadasVliegenthart, HansKrock, BerndVodnar, Dan CristianKrol, EwelinaVohník, MartinKubala, LukášVon Knethen, AndreasKubanek, JuliaVonshak, AvigadKubicova, LenkaVorob’ev, Mikhail M.Kubiński, KonradVougogiannopoulou, KonstantinaKubo, TaiVrontaki, EleniKubota, TakaakiVynios, DemitriosKudo, FumitakaWaheed, Abdul A.Kudryavtsev, Denis S.Walker, Mary K.Kulbacka, JulitaWang, Shin-WeiKulkarni, KetavWase, NishikantKulminskaya, AnnaWatanabe, KenshiKumagai, YuyaWatanabe, RyuichiKumar, ManishWeaver, KeithKumar, RajWee, JosephineKumar, SujeetWeigmann, BennoKung, Chin-PingWeiland-Bräuer, NancyKünzler, MarkusWeiss, TaylorKurihara, HideyukiWelling, MickKusano, ShuheiWells, James W.Kusaykin, Mikhail I.Welsh, MichaelKuyukina, Maria S.Wen, Chiu-MingKuzuya, AkinoriWen, JianjunKwak, Chung ShilWerner, JohannesKwan, JasonWheeler, ThomasLa Clair, JamesWibowo, DavidLa Regina, GiuseppeWibowo, MarioLaatsch, HartmutWie, Myung-BokLabbozzetta, ManuelaWiedman, GregoryLackner, GeraldWielgosz-Collin, GaetaneLafond, MickaëlWijeyeskere, SanjeevaLai, Pin-KuangWiktorska, KatarzynaLaity, Peter RWilkens, CasperLamberto, MassimilianoWilliams, KatherineLane, Katie E.Williams, PhilipLapid, KfirWilliamson, AdeleLaraia, LucaWilson, DavidLatek, DorotaWinchester, LeeLauder, BobWink, JoachimLauersen, KyleWińska, KatarzynaLaurent, FERRIÉWnuk, MaciejLaurienzo, PaolaWojtkielewicz, AgnieszkaLauritano, ChiaraWong, Boon SengLavandera, Jose LuisWong, Clarence T. T.Law, Brian K.Wrigley, StephenLayek, BuddhadevWu, BinLazzara, GiuseppeYagüe, PaulaLe Pogam, PierreYahia, MassinissaLe, Hoang-ThanhYamada, TakeshiLeal, FernandoYamamoto, NorioLebedyeva, IrynaYamamura, SoichiroLee Chang, Kim JyeYamanaka, HidenoriLee, Chang-WooYamashita, AtsushiLee, HyeyoungYaoita, YasunoriLee, Sang KookYasuike, MotoshigeLee, Seung-HongYates, Edwin AlexanderLee, VictorYatsunami, RieLee, Yeon-JuYee, LachlanLeitão, Jorge H.Yeo, SynLemos, Manuel L.Yermak, Irina M. Leon, FranciscoYi, MyunggiLeón, RosaYi, Young-SuLephart, Edwin D.Yokosuka, AkihitoLes, FranciscoYokoya, MasashiLeung, IvanhoeYoshikazu, NakamuraLeung, Yu minYoung, Howard A.Leverett, BetsyYoung, Jette FeveileLewinska, AnnaYurchenko, AntonLewis, RichardZaccaroni, AnnalisaLi, GuijieZafar, Muhammad SohailLi, Hsing-HuiZaitsev, AlekseyLi, MiaomiaoZalubovskis, RaivisLi, Sing-ChungZamyatnin, AndreyLi, XuwenZanolla, MarianelaLiagre, BertrandZárate, RafaelLiaw, Chya-YanZardoya, RafaelLicciardello, GraziaZdzisińska, BarbaraLicea, AlexeiZeidi, MahdiLin, Wei-YongZelepuga, ElenaLinares, Sara GarcíaZhao, XiuboLindstrom, Jon MartinZhao, Xue ZhiLiu, LanZhbannikov, IlyaLiu, Shing-HwaZhou, ShengbinLiu, XiaoxiZhukova, NataliaLiu, YaminZhuravleva, Olesya I.Liu, YangZidovec Lepej, SnjezanaLiverani, ElisabettaZielenkiewicz, UrszulaLlamas, InmaculadaZienkiewicz, KrzysztofLo Giudice, AngelinaZimoch-Korzycka, AnnaŁodyga-Chruścińska, ElżbietaŻołnowska, BeataLokki, InkeriZorov, Dmitry BLopes, GracilianaZubía, EvaLópez-Maury, LuisŽuvela, Petar

